# CT-based radiomics for predicting the treatment response to PD-1/PD-L1 inhibitors combined with chemotherapy in unresectable gastric cancer

**DOI:** 10.1186/s13244-026-02214-7

**Published:** 2026-03-12

**Authors:** Peng-chao Zhan, Shuo Yang, Li-ming Li, Xing Liu, Zhen Cheng, Yu-yuan Zhang, Jia-xing Wang, Qing-liang Chen, Jian-bo Gao

**Affiliations:** 1 Department of Radiology, The Third People’s Hospital of Henan Province, Zhengzhou, PR China; 2 Department of Radiology, The Second Hospital, Cheello College of Medicine, Shandong University, Jinan, PR China; 3 Department of Radiology, The First Affiliated Hospital of Zhengzhou University, Zhengzhou, PR China; 4 Department of Interventional Radiology, The First Affiliated Hospital of Zhengzhou University, Zhengzhou, PR China; 5 Department of Interventional Medicine, The Second Hospital, Cheello College of Medicine, Shandong University, Jinan, PR China

**Keywords:** Gastric cancer, Radiomics, Immunotherapy, Immune infiltration

## Abstract

**Objective:**

To develop and validate a CT-based radiomics model to predict immunotherapy response in unresectable gastric cancer and explore its underlying biological mechanisms.

**Materials and methods:**

This retrospective study included 368 unresectable gastric cancer patients receiving programmed death-1/programmed death ligand-1 (PD-1/PD-L1) inhibitors combined with chemotherapy from two centers. Patients were divided into training (*n* = 231), internal validation (*n* = 97), and external validation (*n* = 40) cohorts. Radiomics model was constructed using portal venous phase CT images, and a radiomics score (Radscore) was calculated for each patient. Five machine learning models incorporating clinical factors and Radscore were developed and compared. The best-performing model was used to construct a nomogram. Model performance was assessed using the area under the receiver operating characteristic curve (AUC), calibration curves, and decision curve analysis (DCA). Immune cell infiltration analysis was performed using data from The Cancer Genome Atlas (TCGA) cohort.

**Results:**

The radiomics signature, comprising 15 selected features, showed good predictive performance across all cohorts: training (AUC = 0.868), internal validation (AUC = 0.816), and external validation (AUC = 0.793). The logistic regression model demonstrated the highest and most consistent performance, with AUC values of 0.886, 0.831, and 0.826, respectively. The developed nomogram showed satisfactory calibration and clinical utility. Immune infiltration analysis revealed significant associations between Radscore and infiltration levels of activated CD4+ memory T cells, regulatory T cells, and CD8+ T cells.

**Conclusions:**

The CT-based radiomics nomogram showed promise for personalizing immunotherapy treatment strategies in unresectable gastric cancer. The association between the Radscore and immune cell infiltration provided insights into its biological basis.

**Critical relevance statement:**

This rigorously validated CT radiomics nomogram critically advances gastric cancer immunotherapy prediction, offering clinical radiology a non-invasive, biologically-informed tool to guide personalized treatment decisions.

**Key Points:**

- CT radiomics provided a reliable marker for predicting gastric cancer immunotherapy response.
- The developed Radscore correlated with immune cell infiltration, offering biological insights.
- A nomogram integrating the Radscore and clinical factors showed robust predictive performance.

**Graphical Abstract:**

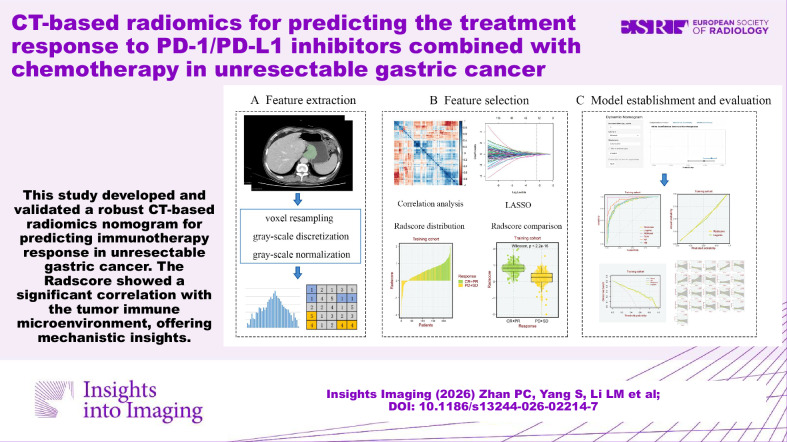

## Introduction

Gastric cancer remains a significant global health burden, ranking as the fifth most prevalent malignancy and the fourth leading cause of cancer-related mortality worldwide [1]. While surgical intervention offers favorable prognoses for early-stage disease, a substantial majority (60–70%) of patients present at advanced stages where curative resection is unfeasible [2, 3]. For unresectable advanced gastric cancer, traditional chemotherapy regimens offer limited survival benefits, with median overall survival often falling below 1 year [4, 5]. The advent of immunotherapy, particularly PD-1/PD-L1 inhibitors, has genuinely revolutionized the therapeutic landscape [6]. Notably, trials like KEYNOTE-062 have demonstrated significant survival extensions when pembrolizumab is combined with chemotherapy in PD-L1-positive advanced gastric cancer patients [7]. Consequently, this combination has become a recommended first-line treatment in major clinical guidelines [8–10].

Despite this progress, accurately predicting treatment response to this combination therapy remains a critical clinical challenge. Current predictive biomarkers, such as PD-L1 expression and microsatellite instability (MSI) status, necessitate invasive biopsies or surgical specimens [11]. These procedures are inherently limited by issues of tumor heterogeneity, lengthy processing times, and considerable costs, thereby hindering their widespread adoption and utility in routine clinical practice [12, 13]. Therefore, developing non-invasive, reliable, and cost-effective predictive tools for identifying patients most likely to benefit from this combination therapy is crucial.

Radiomics, a technique for extracting quantifiable data from medical images, has emerged as a promising non-invasive approach to assess tumor characteristics and predict treatment outcomes [14, 15]. Recent studies have begun to explore the potential of CT radiomics in characterizing the tumor-immune microenvironment of gastric cancer and forecasting immunotherapy efficacy [16–20]. Some investigations have focused on indirectly predicting immunotherapy response by correlating radiomics features with specific immune components, such as CD8+ T cell infiltration or the neutrophil-to-lymphocyte ratio (NLR) [16, 17]. However, such indirect approaches may inherently fail to capture the full complexity of tumor-immune interactions, and their validation has often been limited to smaller immunotherapy cohorts, potentially constraining predictive accuracy. Other investigations have directly employed CT radiomics to predict immunotherapy outcomes [19, 20], but these studies were constrained by small sample sizes and lacked an exploration of the biological underpinnings that could explain the predictive radiomics features. Crucially, research specifically dedicated to predicting the response to the now-standard regimen of PD-1/PD-L1 inhibitors combined with chemotherapy remains notably sparse.

Therefore, this study aims to develop and validate a CT-based radiomics model for predicting treatment response to this standard-of-care combination therapy in unresectable gastric cancer. Furthermore, we will investigate the relationship between radiomics features and the tumor-immune microenvironment, elucidating the biological mechanisms underlying its predictive power for immunotherapy response.

## Materials and methods

This retrospective two-center study received approval from the local ethics committee (2021-KY-1070-002), and the requirement for informed written consent was waived.

### Patient selection

**Fig. 1:**
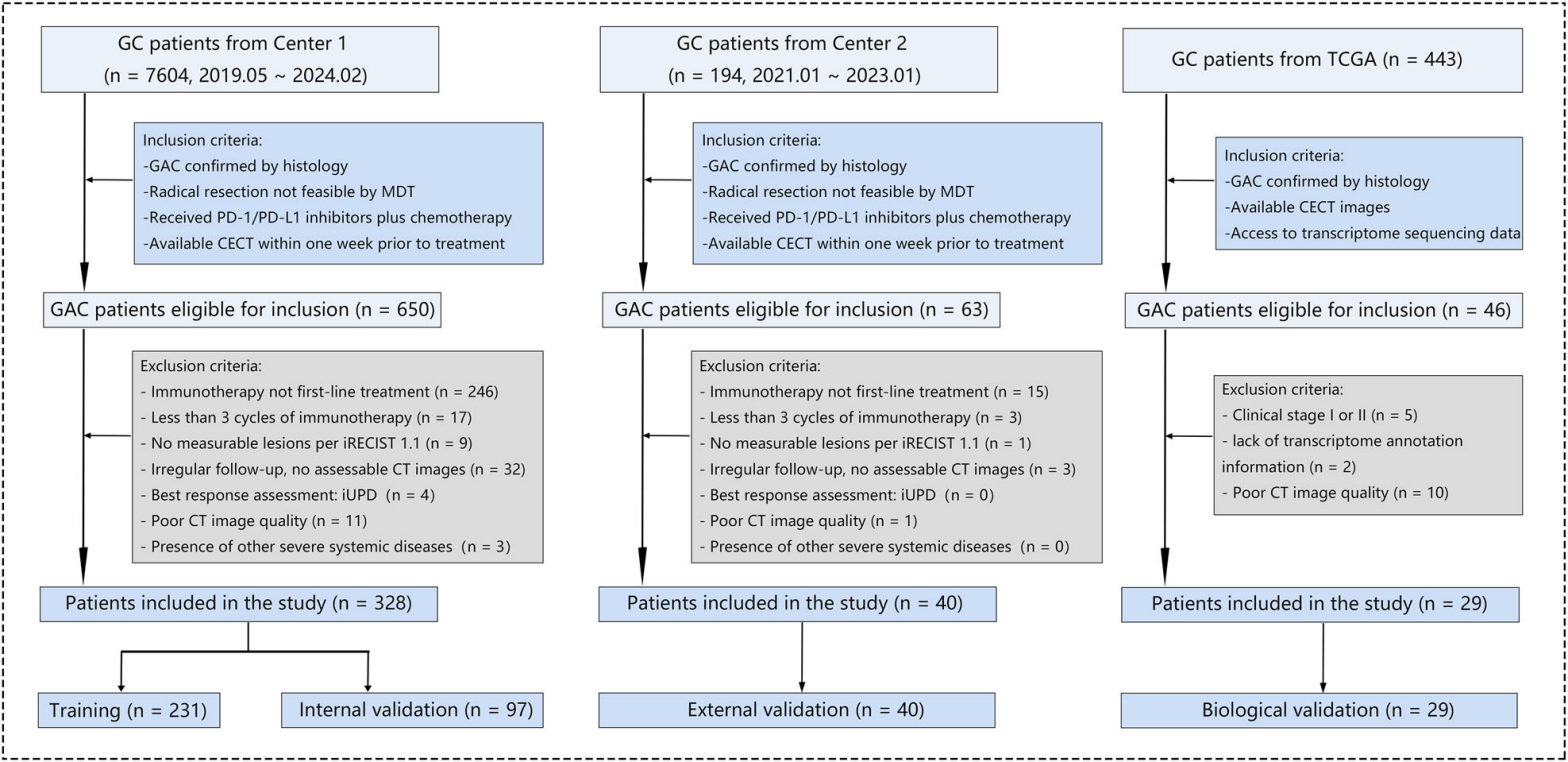
Flowchart of enrolled patients

We conducted a retrospective analysis of clinical data from 7604 consecutive patients at Center 1 (The First Affiliated Hospital of Zhengzhou University) between May 2019 and February 2024. Following the inclusion and exclusion criteria specified in Text S1, we identified 328 eligible patients for further analysis. These patients were subsequently divided into a training cohort (*n* = 231) and an internal validation cohort (*n* = 97) through stratified random sampling in a 7:3 ratio. Additionally, an external validation cohort was comprised of 40 patients from Center 2 (The Second Hospital of Shandong University) during the period from January 2021 to January 2023. The details of the patient recruitment process are depicted in Fig. 1.

To investigate the biological interpretability of the radiomics model from the perspective of the immune microenvironment, we performed an analysis using data from 443 gastric cancer patients included in the TCGA-STAD dataset of The Cancer Genome Atlas (TCGA). Clinical and transcriptomic data were acquired from the TCGA official website (https://portal.gdc.cancer.gov/), while corresponding CT images were sourced from The Cancer Imaging Archive (TCIA, https://www.cancerimagingarchive.net/). The inclusion and exclusion criteria applied to this cohort are comprehensively outlined in Text S1, ultimately leading to the selection of 29 patients for the study.

### Immunotherapy regimens and response assessment

The details of the immunotherapy regimens are described in Text S2. The treatment response was evaluated using the immune Response Evaluation Criteria in Solid Tumors (iRECIST) [21]. The iRECIST criteria encompass immune complete response (iCR), immune partial response (iPR), immune stable disease (iSD), and immune confirmed progressive disease (iCPD). Patients who achieved iCR or iPR were classified into the treatment response group, whereas those exhibiting iSD or iCPD were categorized into the non-response group.

### CT image acquisition and analysis

CT imaging protocol and parameters are detailed in Table S1. L.X. and L.L., possessing 10 and 6 years of experience, respectively, in gastrointestinal tumor imaging, conducted independent evaluations of CT imaging features. For qualitative assessments, any initial discrepancies were resolved through a joint review to reach a consensus. A senior radiologist (G.J., with 40 years of experience) was available to arbitrate any unresolved disagreements. For quantitative measurements, the values from the two observers were averaged. The observers were informed that the study pertained to gastric cancer patients but remained blinded to additional clinical and treatment details. The following CT image features were evaluated: (1) tumor location; (2) tumor size; (3) CT values; (4) enhancement degree; (5) enhancement pattern; and (6) clinical staging. A more detailed description of the evaluation criteria for these features is provided in Text S3.

### Clinicoradiological features associated with treatment response

A two-stage process was employed to identify clinical independent predictors of treatment response using the training cohort data. First, a univariate logistic regression analysis was performed for each clinical and CT feature to assess their individual association with treatment response. In the second stage, variables showing a promising association in the univariate analysis (defined by a *p* < 0.2) were advanced for multivariate analysis. Variables remaining in the final model with a *p* < 0.05 were considered statistically significant independent predictors of treatment response.

### Radiomics feature extraction and selection

The details of image processing and region of interest (ROI) segmentation are provided in Text S4. Based on each manually delineated ROI, we extracted 851 radiomics features, which included 107 original features and 744 wavelet-transformed features. The reliability of the radiomics features was evaluated using inter- and intra-class correlation coefficients (ICCs). For the assessment of inter-class consistency, LP and LL independently delineated ROIs and extracted features from a cohort of 30 randomly selected patients from the training cohort. To evaluate intra-class consistency, LL re-delineated the ROIs and re-extracted the features following a 2-week interval.

Utilizing the training cohort, we performed dimensionality reduction on the extracted features to identify an optimal subset for constructing the radiomics signature. This process involved the following steps: first, we retained features with ICCs ≥ 0.75 for further analysis; next, we removed redundant features exhibiting a Pearson correlation coefficient of 0.95 or higher, and finally, we employed the least absolute shrinkage and selection operator (LASSO) method with 10-fold cross-validation.

### Models construction and evaluation

A clinical model was developed using the logistic regression algorithm based on clinical independent predictors. The radiomics score (Radscore) was computed utilizing the selected features and their weighted coefficients. Furthermore, five machine learning algorithms were employed to construct the combined model integrated independent predictors and Radscore: logistic regression, eXtreme Gradient Boosting (XGB), support vector machine (SVM), naive Bayesian (NB), and random forest (RF). The hyperparameter settings of the machine learning models and the R packages used have been detailed in Text S5.

The efficacy of models was evaluated by the area under the curve (AUC) of the receiver operating characteristic (ROC) curve. AUC values of different models were compared by DeLong test. Based on the AUC values and the results of the DeLong test, the optimal machine learning model was identified, leading to the development of a nomogram. Calibration curves were utilized to verify the concordance between predicted and observed probabilities, and the Hosmer-Lemeshow goodness-of-fit test was conducted to evaluate the potential for overfitting. Additionally, decision curve analysis (DCA) was performed to determine the clinical utility of the models.

### Immune cell infiltration assessment

For the 29 patients in the TCGA cohort, ROIs were delineated by LL following the same criteria used for the primary cohorts. The radiomics features were then extracted, and the Radscore for each patient was calculated based on the radiomics signature derived from the training cohort. Utilizing RNA sequencing data, we employed the IOBR package to quantitatively evaluate the abundance of 22 distinct immune cell types, encompassing T cells, B cells, and macrophages. To elucidate the biological basis of the radiomics signature, we performed a Spearman’s rank correlation analysis between each patient’s Radscore with the abundance of the 22 quantified immune cell types.

### Statistical analysis

Statistical analyses were performed using R software (version 4.2.2). The Shapiro–Wilk test assessed the normality of quantitative data. Normally distributed variables were expressed as mean ± SD, while non-normal data were presented as median (interquartile range). Independent-sample *t*-tests or Mann–Whitney U tests compared treatment response groups. Categorical variables were described as frequencies and analyzed with Pearson’s chi-squared test, continuity correction, or Fisher’s exact test.

## Results

### Baseline characteristics

A total of 368 patients diagnosed with gastric adenocarcinoma were included in this study. Table 1 provides a summary of the baseline characteristics of patients. No significant differences were observed in the baseline clinical characteristics among the three cohorts (all *p* > 0.05).

**Table 1:** Baseline characteristics of patients

Characteristic	Training (*n* = 231)	Internal validation (*n* = 97)	External validation (*n* = 40)	Statistics	*p*-value
Age (years)	62.29 ± 12.09	61.43 ± 11.90	60.68 ± 9.99	0.413	0.662^a^
Gender (*n*, %)				3.618	0.164^b^
Male	170 (73.6%)	65 (67.0%)	33 (82.5%)		
Female	61 (26.4%)	32 (33.0%)	7 (17.5%)		
Clinical stage (*n*, %)				2.038	0.361^b^
III	59 (25.5%)	32 (33.0%)	10 (25.0%)		
IV	172 (74.5%)	65 (67.0%)	30 (75.0%)		
Treatment cycles	6.23 ± 2.82	6.26 ± 2.71	6.78 ± 3.10	0.668	0.519^a^
Efficacy (*n*, %)				0.561	0.756^b^
Response	119 (51.5%)	50 (51.6%)	18 (45.0%)		
Non-response	128 (48.5%)	47 (48.4%)	22 (55.0%)		

^a^ *t*-tests

^b^ Chi-squared test

### Clinicoradiological features associated with treatment response

The results of the logistic regression analyses are presented in Table 2. In the initial univariate screening, several variables met the pre-specified inclusion criterion of *p* < 0.2 for further analysis. These candidate predictors included treatment cycles, tumor location, portal venous phase CT value, platelets, neutrophils, the platelet-to-lymphocyte ratio and CA72-4 status. After adjusting for these variables in the multivariate logistic regression model, only two factors emerged as statistically significant independent predictors of treatment response: treatment cycles (OR = 1.20; 95% CI: 1.07–1.35; *p* = 0.001) and CA72-4 status (OR = 0.50; 95% CI: 0.28–0.91; *p* = 0.024). These variables were subsequently used to construct a clinical model.

**Table 2:** Results of logistic regression analysis in the training cohort

Characteristic (reference)	Univariate			Multivariate		
	OR	95% CI	*p*-value	OR	95% CI	*p*-value
Age	1.01	0.99–1.03	0.485			
Gender (male)	0.88	0.49–1.58	0.671			
Treatment cycles	1.23	1.1–1.37	**< 0.001**	1.20	1.07–1.35	**0.001**
Tumor location (cardia)						
Body	0.5	0.26–0.98	**0.042**	0.51	0.25–1.03	0.062
Antrum	0.44	0.21–0.9	**0.024**	0.51	0.23–1.11	0.088
≥ 2/3 stomach	0.61	0.27–1.36	0.227	0.57	0.24–1.37	0.208
Plain scan CT value	1	0.97–1.03	0.931			
Arterial phase CT value	0.99	0.98–1.01	0.375			
Portal venous phase CT value	0.99	0.98–1	**0.056**	0.99	0.98–1	0.165
Enhancement degree (mild to moderate)	1	0.59–1.68	1			
Enhancement pattern (persistent)	/	/	/			
Washout	1.11	0.49–2.53	0.797			
Progressive	0.76	0.34–1.72	0.513			
Tumor length	1	0.99–1.01	0.520			
Tumor thickness	1.02	0.99–1.05	0.201			
Clinical T stage (T3)	1.08	0.58–2.02	0.809			
Clinical N stage (N0)	1.36	0.7–2.66	0.369			
Clinical M stage (M0)	1.28	0.75–2.17	0.364			
Clinical stage (III)	/	/	/			
IV	1.24	0.30–5.12	0.470			
Hemoglobin	0.99	0.98–1	0.228			
Platelets	1	1–1.01	**0.118**	1	1–1.01	0.503
Neutrophils	1.14	0.97–1.34	**0.117**	1.1	0.91–1.33	0.309
Lymphocytes	1.11	0.67–1.86	0.681			
Monocytes	1.07	0.23–4.9	0.936			
NLR	1.07	0.93–1.22	0.351			
PLR	1.00	1–1	**0.198**	1.00	1–1	0.59
LMR	0.97	0.84–1.12	0.647			
CEA (normal)	1.23	0.73–2.08	0.431			
CA19-9 (normal)	0.88	0.5–1.55	0.657			
CA72-4 (normal)	0.62	0.36–1.07	**0.087**	0.50	0.28–0.91	**0.024**

*NLR* neutrophil-to-lymphocyte, *PLR* platelet-to-lymphocyte, *LMR* lymphocyte-to-monocyte, *CEA* carcinoembryonic antigen, *CA19-9* carbohydrate antigen 19-9, *CA72-4* carbohydrate antigen 72-4

The bold *p*-values in the univariate analysis were to highlight variables with *p*-values less than 0.2 for further analysis. In the multivariate analysis, the bold *p*-values were used to emphasize variables with final *p*-values less than 0.05

### Radiomics signature construction

**Fig. 2:**
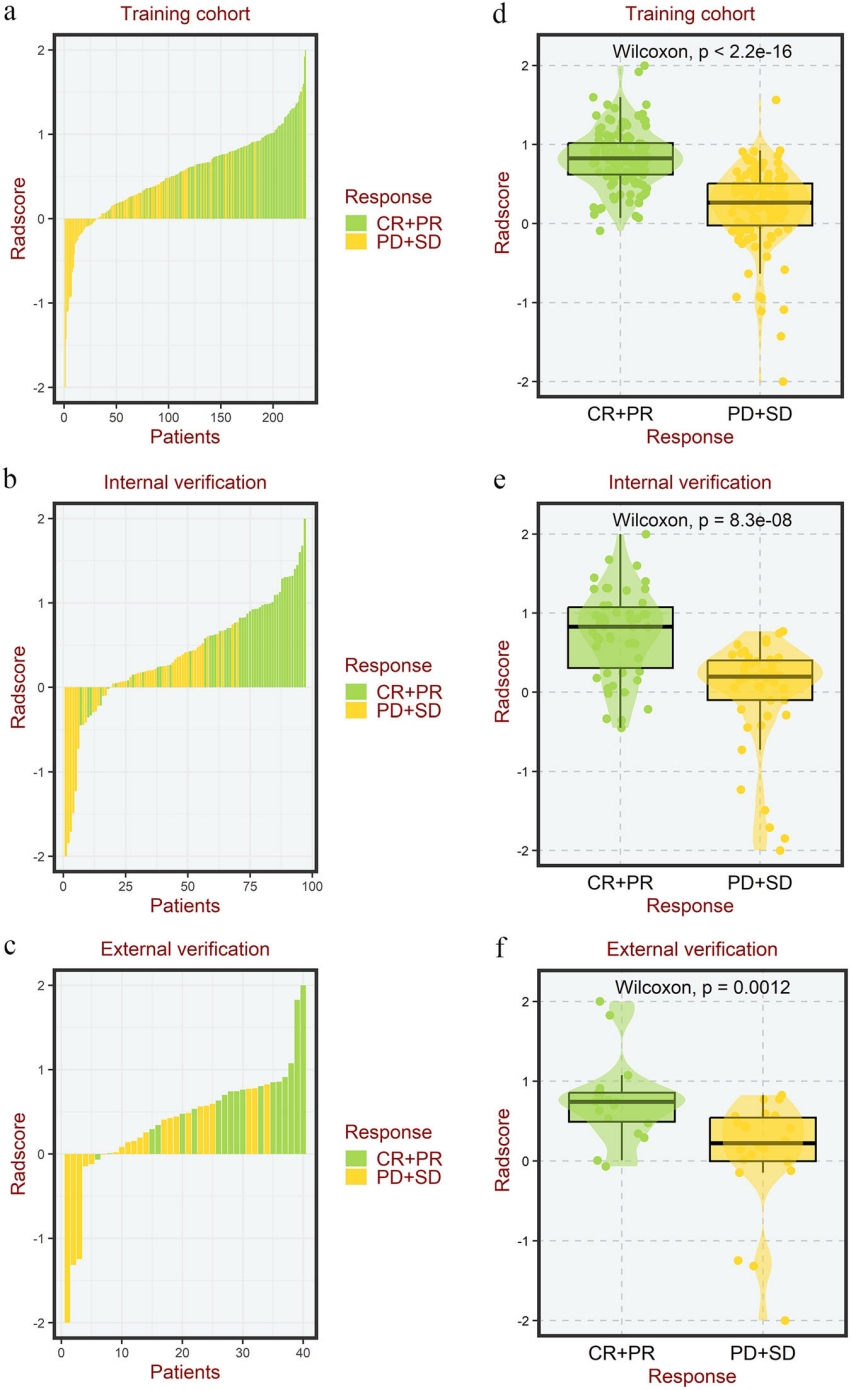
Distribution of Radscore among gastric cancer patients across different cohorts, alongside a comparative analysis of Radscore between response and non-response groups. **a**, **d** The training cohort; **b**, **e** the internal validation cohort; **c**, **f** the external validation cohort

A total of 439 features with low ICCs or high inter-feature correlation were removed, leaving 412 features for analysis. LASSO regression identified 15 optimal radiomics features for constructing the radiomics signature (Fig. S1, Table 3). The distribution of Radscore across each cohort is illustrated in Fig. 2. Notably, the Radscore was significantly elevated in the treatment response group compared to the non-response group across all three cohorts (*p* < 0.001 for all comparisons) (Fig. 2).

**Table 3:** Radiomics signature

Features	Coefficient
Intercept	0.049124367
Original shape Flatness	−0.108127438
Wavelet LLH glcm Imc1	−0.00194955
Wavelet LLH ngtdm Busyness	−0.011553971
Wavelet LHL glcm Autocorrelation	−0.023343715
Wavelet LHH firstorder Kurtosis	0.070103348
Wavelet HLL glcm MaximumProbability	0.032363627
Wavelet HLL glszm GrayLevelNonUniformity	−0.271672778
Wavelet HLL ngtdm Contrast	−0.237148842
Wavelet HLH firstorder Kurtosis	0.205449843
Wavelet HLH firstorder Median	0.262356661
Wavelet HHL glcm ClusterShade	−0.073857162
Wavelet HHL glcm InverseVariance	0.024585599
Wavelet HHL glszm GrayLevelVariance	−0.022673688
Wavelet LLL firstorder Maximum	−0.062524036
Wavelet LLL glszm GrayLevelNonUniformityNormalized	0.187431418

### Models construction and performance comparison

The performance metrics for each model, including AUC, accuracy, sensitivity, and specificity, are presented in Table 4. The performance of the Radscore, with AUCs of 0.868, 0.816, and 0.793 in the training, internal validation, and external validation cohorts, respectively, was substantially superior to that of the clinical model (AUCs of 0.538, 0.571, and 0.535, respectively; DeLong test, all *p* < 0.05 as detailed in Table S2).

**Table 4:** Diagnostic performance of different machine learning models

Models	Datasets	Diagnostic performance			
		AUC (95% CI)	Accuracy	Sensitivity	Specificity
Clinical	Training	0.538 (0.463–0.612)	55.8%	83.2%	26.8%
	Internal validation	0.571 (0.456–0.685)	57.7%	32.0%	85.1%
	External validation	0.535 (0.344–0.727)	62.5%	55.6%	68.2%
Radscore	Training	0.868 (0.823–0.913)	80.1%	78.2%	82.1%
	Internal validation	0.816 (0.728–0.904)	80.4%	70.0%	91.5%
	External validation	0.793 (0.650–0.936)	77.5%	66.7%	86.4%
Logistic	Training	0.886 (0.844–0.928)	81.8%	79.0%	84.8%
	Internal validation	0.831 (0.749–0.913)	79.4%	70.0%	89.4%
	External validation	0.826 (0.684–0.967)	80.0%	66.7%	90.9%
XGB	Training	0.991 (0.979–1.00)	99.1%	99.2%	99.1%
	Internal validation	0.760 (0.662–0.857)	72.2%	50.0%	95.7%
	External validation	0.707 (0.540–0.874)	70.0%	88.9%	54.5%
SVM	Training	0.887 (0.844–0.929)	83.5%	82.4%	84.8%
	Internal validation	0.801 (0.707–0.895)	80.4%	68.0%	93.6%
	External validation	0.702 (0.536–0.868)	72.5%	66.7%	77.3%
RF	Training	0.939 (0.910–0.967)	86.1%	80.7%	92.0%
	Internal validation	0.825 (0.739–0.910)	79.4%	76.0%	83.0%
	External validation	0.799 (0.645–0.954)	82.5%	61.1%	100.0%
NB	Training	0.868 (0.821–0.915)	80.5%	80.7%	80.4%
	Internal validation	0.823 (0.739–0.908)	79.4%	70.0%	89.4%
	External validation	0.760 (0.608–0.913)	72.5%	83.3%	63.6%

*XGB* eXtreme Gradient Boosting, *SVM* support vector machine, *NB* naive Bayesian, *RF* random forest

**Fig. 3:**
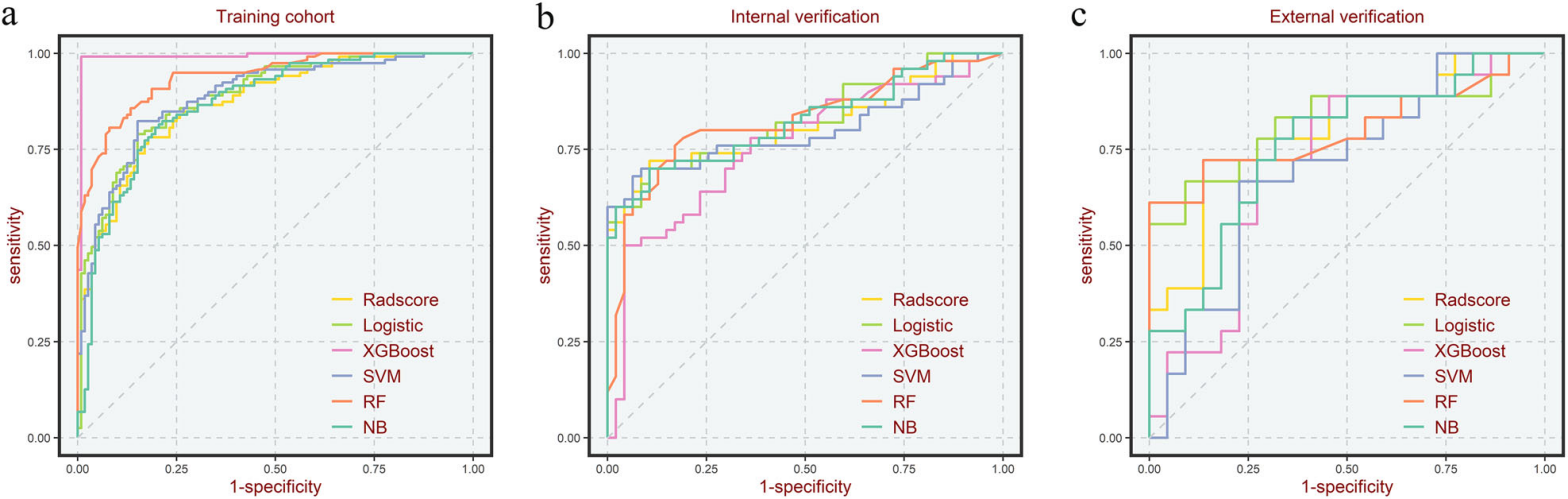
Receiver operating characteristic curves (ROC) for different models. **a** The training cohort; **b** the internal verification cohort; **c** the external verification cohort

A combined model was subsequently developed using five machine learning methods, and a detailed comparison of their performance is presented in Table 4, with corresponding ROC curves depicted in Fig. 3. In the training cohort, the XGBoost model exhibited the highest AUC (0.991), but it demonstrated substantial performance fluctuations of 23.3% and 28.7% in the internal and external validation cohorts. In contrast, the logistic regression model achieved the highest and most stable AUC values in both the internal validation (0.831) and external validation (0.826) cohorts, with minimal AUC variations of only 6.2% and 6.8%, respectively. The full results of the DeLong tests comparing all models are presented in Table S2, confirming the logistic regression combined model as the optimal choice based on its balance of high accuracy and stability.

### Nomogram construction and evaluation

**Fig. 4:**
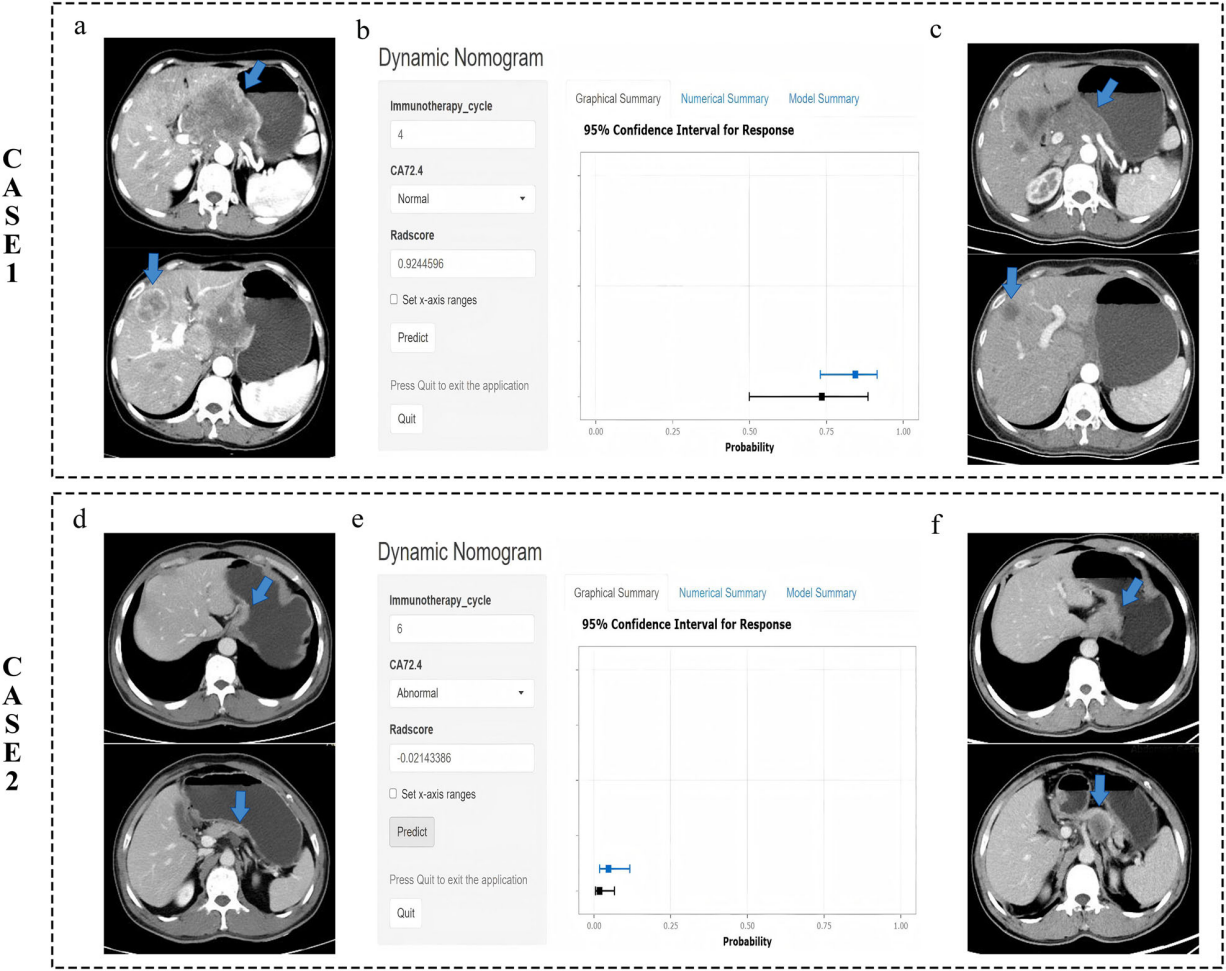
Examples of clinical application of the dynamic nomogram to predict treatment response to PD-1/PD-L1 inhibitors combined with chemotherapy in gastric cancer. Case 1: A 53-year-old female patient with stage IV gastric adenocarcinoma, normal CA72-4 status, and a Radscore of 0.9244596. **a** Baseline contrast-enhanced CT showed a mass in the cardia and lesser curvature of the stomach (arrow), with a visible intrahepatic metastasis (arrow). **b** Inputting the CA72-4 status (normal) and Radscore (0.9244596) into the dynamic nomogram yielded a predicted response probability of 73.5% at treatment cycle 0, which increased to 84.4% at cycle 4. **c** The patient received 4 cycles of combination therapy. Follow-up contrast-enhanced CT revealed significant shrinkage of both the primary gastric mass and the intrahepatic metastasis. Case 2: A 55-year-old male patient with stage IV gastric adenocarcinoma, abnormal CA72-4 status, and a Radscore of −0.02143386. **d** Baseline contrast-enhanced CT showed thickening of the gastric wall at the cardia (arrow) and an enlarged lymph node in the gastrohepatic space (arrow). **e** Inputting the CA72-4 status (abnormal) and Radscore (−0.02143386) into the dynamic nomogram yielded a predicted response probability of 1.8% at treatment cycle 0, which increased to 4.7% at cycle 6. **f** The patient received 6 cycles of combination therapy. Follow-up contrast-enhanced CT revealed significant enlargement of the cardiac lesion and the gastrohepatic lymph node

The logistic regression combined model was selected to construct a dynamic nomogram for the visualization and clinical prediction of treatment response. The final model is defined by the following equation: Logit(*p*) = −2.97815 + 0.1672362 × treatment cycles − 0.9394547 × CA72-4 + 4.3234098 × Radscore (Note: *p* represents the probability of achieving a treatment response; CA72-4 is a dichotomous variable where 0 = normal and 1 = abnormal). Furthermore, to maximize its accessibility and ease of use, this nomogram has been deployed as an interactive web application, publicly available at: https://zpc0v0.shinyapps.io/DynNomapp/. This online tool enables clinicians to input individual patient data (treatment cycles, CA72-4 status, and Radscore) to obtain an immediate, personalized prediction of immunotherapy response probability (Fig. 4).

**Fig. 5:**
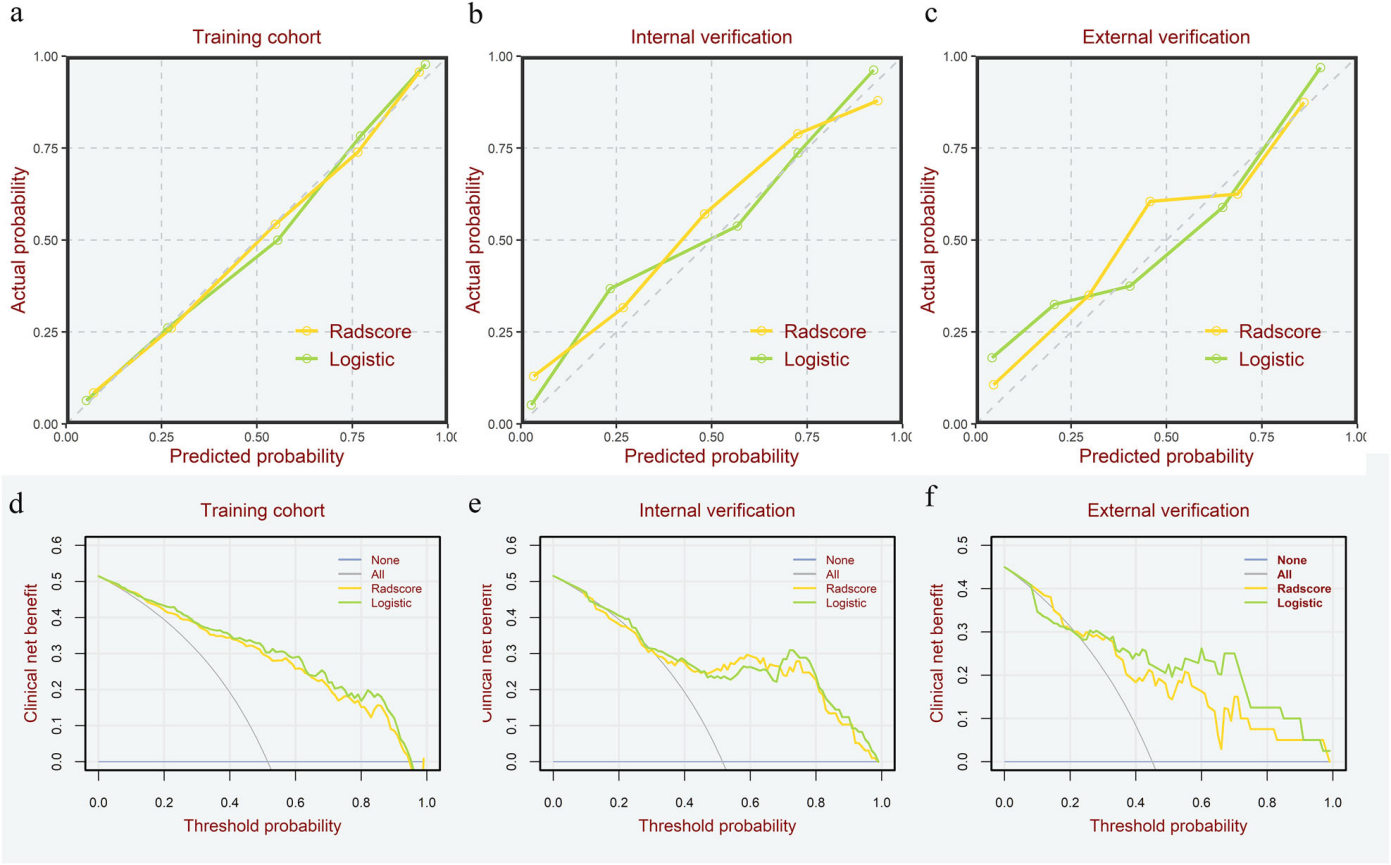
Calibration curves and decision curves for both the radiomics and logistic regression models. **a**, **d** The training cohort; **b**, **e** the internal validation cohort; **c**, **f** the external validation cohort

We then evaluated the calibration and clinical utility of the final nomogram and the radiomics-only model. Calibration curves indicated that the predictions generated by both models were well-aligned with actual outcomes in evaluating the response, demonstrating strong consistency. The Hosmer-Lemeshow test results revealed no significant differences between predicted and observed outcomes (all *p* > 0.05), suggesting a good model fit (Fig. 5). DCA curves further demonstrated that utilizing predictions from both models to guide clinical interventions yielded net clinical benefits for patients over a broad range of threshold probabilities (Fig. 5).

### Immune infiltration analysis

**Fig. 6:**
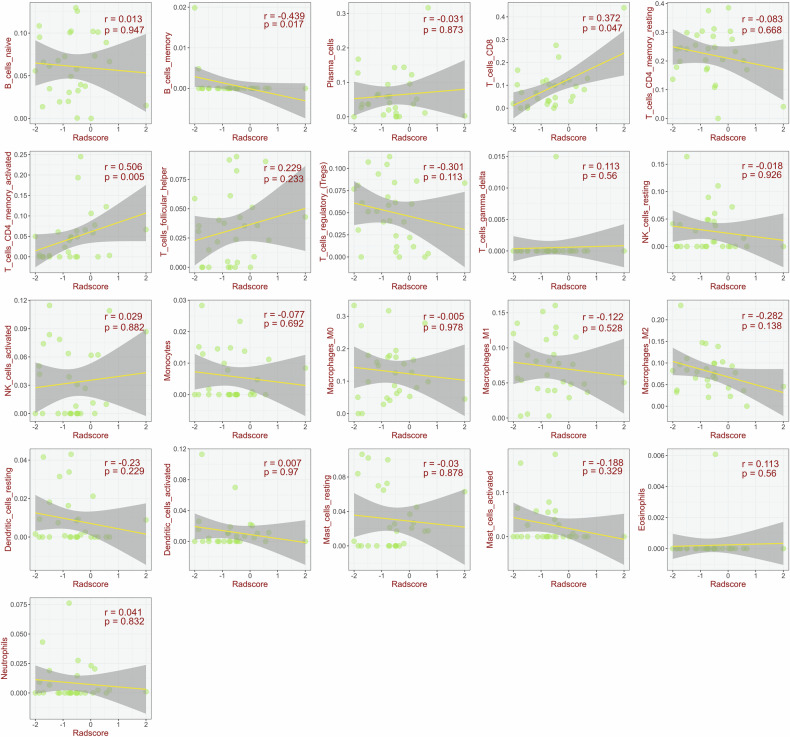
Results of Spearman’s rank correlation analysis. The horizontal axis represents the Radscore, and the vertical axis represents the different types of immune cells

The median Radscore for the 29 patients within the TCGA cohort was determined to be −0.71, with an interquartile range of −1.18 to −0.20. Spearman’s rank correlation analysis (Fig. 6) revealed that Radscore was positively correlated with activated CD4+ memory T cells (*r* = 0.506, *p* = 0.005) and CD8+ T cells (*r* = 0.372, *p* = 0.047), while it was negatively correlated with B memory cells (*r* = −0.439, *p* = 0.017).

## Discussion

This study successfully developed and validated a CT-based radiomics nomogram that demonstrated robust predictive performance and generalizability for predicting immunotherapy response in unresectable gastric cancer. Importantly, the Radscore was significantly associated with the tumor-immune microenvironment, providing a biological basis for its predictive power and a theoretical foundation for its clinical application in guiding treatment decisions.

While conventional clinical and radiological features are integral to unresectable gastric cancer management, their utility in predicting immunotherapy efficacy is often limited. Previous research has largely focused on correlating biomarkers like MSI and PD-L1 status with clinicopathological characteristics [22–25] or analyzing the impact of clinical factors on immunotherapy outcomes [26]. For instance, studies have linked the number of metastatic organs [27] and peripheral blood biomarkers such as NLR [17] to immunotherapy efficacy. Others have shown that age, tumor stage, and tumor markers such as CA125 and CA 72-4 are significant predictors of postoperative adjuvant immunotherapy prognosis [28]. Emerging imaging techniques like dual-energy CT offer novel parameters, such as iodine uptake, which have shown promise in predicting immunotherapy response [29, 30]. In this study, although treatment cycles and CA72-4 levels emerged as independent prognostic factors in multivariate analysis, incorporating them into the radiomics model did not significantly improve predictions. This highlights the limitations of conventional clinical features in capturing tumor heterogeneity and predicting individual treatment responses. Future models should integrate comprehensive clinical parameters and multi-modal imaging features for more precise and personalized treatment decisions.

In recent years, CT-based radiomics has emerged as a valuable non-invasive tool for assessing tumor heterogeneity and predicting immunotherapy outcomes in unresectable gastric cancer, offering a more comprehensive approach than traditional methods. Studies by Sun et al [16] and Huang et al [17] have demonstrated the potential of radiomics to characterize the tumor-immune microenvironment by predicting CD8+ cell infiltration and intratumoral NLR levels, respectively, which correlated with immunotherapy response. Furthermore, research has directly utilized CT radiomics to predict immunotherapy outcomes. For instance, Liang et al [20] and Huang W et al [31] developed radiomics nomograms and imaging biomarkers predicting response to PD-1 inhibitors and progression-free survival, respectively, with promising AUCs and associations with immune components like M1 macrophage infiltration. Building upon this foundation, our study specifically focused on predicting response to the standard combination of PD-1/PD-L1 inhibitors and chemotherapy. We extracted radiomics features from portal venous phase CT images and utilized the LASSO algorithm to construct a radiomics model significantly correlated with the response to immunotherapy. The results indicated that the Radscore exhibited excellent predictive performance in distinguishing patient groups with varying treatment responses, achieving AUC values of 0.868, 0.816, and 0.793 in three cohorts, respectively. Calibration curves and DCA further substantiated its reliability and clinical applicability. These findings, which align with previous research, suggest that CT radiomics can identify imaging features associated with the response to immunotherapy.

The application of machine learning algorithms is essential in developing robust predictive models from complex radiomic data [32]. In this study, we evaluated five mainstream machine learning algorithms. While most models exhibited good predictive performance in the training cohort (AUC > 0.7), their stability varied. Notably, the XGBoost model, despite achieving the highest AUC in the training cohort (AUC = 0.991), showed a significant performance decline in validation cohorts (AUC = 0.760 and 0.707, respectively), indicating a propensity for overfitting. In contrast, the logistic regression model demonstrated superior generalizability, maintaining consistently high AUC values across all cohorts (0.886, 0.831, and 0.826) with minimal variability. This highlights the critical importance of rigorous model selection and external validation in clinical applications. The logistic regression model’s strong predictive accuracy and clinical utility, further substantiated by calibration curves and DCA, led to its selection for constructing a visual nomogram, providing clinicians with an intuitive tool to aid personalized treatment decisions.

The composition and functional status of the tumor-immune microenvironment play a crucial role in determining immunotherapy outcomes [33]. Key effector cells like CD8+ T cells are associated with favorable responses [34], while CD4+ T cells provide crucial support [35]. Conversely, regulatory T cells (Tregs) often mediate immunosuppression and resistance [36], and B cell roles can be complex [37]. Our analysis of TCGA data revealed that the Radscore positively correlated with activated CD4+ memory T cells (*r* = 0.506, *p* = 0.005) and CD8+ T cells (*r* = 0.372, *p* = 0.047) and negatively with B memory cells (*r* = −0.439, *p* = 0.017). These findings suggest that a high Radscore reflects a more pro-inflammatory, anti-tumor-immune microenvironment, whereas a low Radscore may indicate an immunosuppressive milieu. This implies that the Radscore captures intrinsic TIME characteristics, thereby serving as a marker for immunotherapy sensitivity and offering valuable insights for personalized treatment strategies.

Several limitations should be acknowledged. Firstly, the retrospective nature of this study, along with a relatively limited sample size, may introduce selection bias and affect the generalizability of our findings. Second, while effective, the machine learning algorithms employed did not include more advanced deep learning techniques, which could potentially offer further improvements. Finally, the TCGA/TCIA cohort analysis, also retrospective and based on a modest sample, warrants cautious interpretation and further validation.

In conclusion, we developed and validated a robust CT-based radiomics nomogram to predict immunotherapy response in unresectable gastric cancer. Our findings revealed a significant link between the Radscore and the tumor-immune microenvironment, providing mechanistic insight. Future studies should leverage larger, multi-modal datasets and advanced algorithms to enhance the model’s predictive precision.

## Supplementary information

ELECTRONIC SUPPLEMENTARY MATERIAL

## Data Availability

The dataset used or analyzed during the current study is available from the corresponding author upon reasonable request.

## Abbreviations

AUC: Area under the curve
CI: Confidence interval
DCA: Decision curve analysis
iRECIST: Immune response evaluation criteria in solid tumors
LASSO: Least absolute shrinkage and selection operator method
PD-1: Programmed death-1
PD-L1: Programmed death ligand-1
ROC: Receiver operating characteristic curve
ROI: Region of interest
XGB: eXtreme Gradient Boosting
