# Supplementary material for: CT-based radiomics for predicting the treatment response to PD-1/PD-L1 inhibitors combined with chemotherapy in unresectable gastric cancer
Source: Insights Imaging. 2026 Mar 12;17:66. doi: 10.1186/s13244-026-02214-7 (PMC12982818; doi:10.1186/s13244-026-02214-7)
Supplement: Supplementary file 1 — ELECTRONIC SUPPLEMENTARY MATERIAL [file 13244_2026_2214_MOESM1_ESM.pdf]

CT-based Radiomics for Predicting the Treatment Response to PD-1/PD-L1 Inhibitors Combined with Chemotherapy in Unresectable Gastric Cancer

ELECTRONIC SUPPLEMENTARY MATERIAL

Text S1. Details of inclusion and exclusion criteria

For patients at Centers 1 and 2, the inclusion criteria were as follows: (1) histopathological confirmation of gastric adenocarcinoma (GAC); (2) determination of inoperability for curative resection by a multidisciplinary team; (3) administration of PD-1/PD-L1 inhibitors in conjunction with chemotherapy; and (4) completion of non-contrast and dual-phase contrast-enhanced CT scans within one week prior to the initiation of combined therapy (baseline CT). The exclusion criteria included: (1) prior receipt of any anti-tumor treatments before the combined therapy; (2) completion of fewer than three cycles of the combined therapy; (3) absence of measurable lesions or lack of regular follow-ups necessary for response evaluation; (4) classification of the best overall response as immune unconfirmed progressive disease (iUPD); (5) suboptimal CT image quality or inadequate gastric distension that compromises lesion evaluation and delineation; and (6) presence of concurrent primary malignancies or severe systemic diseases.

The inclusion criteria for the TCGA cohort were as follows: (1) histopathological confirmation of GAC; (2) availability of comprehensive non-contrast and contrast-enhanced CT imaging; and (3) access to transcriptome sequencing data. The exclusion criteria comprised: (1) clinical stage I or II disease; (2) lack of transcriptome annotation information; and (3) suboptimal CT image quality or insufficient gastric distension, which could impede accurate lesion evaluation and delineation.

Text S2. Immunotherapy regimens

All patients diagnosed with gastric cancer were treated with a combination of PD-1/PD-L1 inhibitors and chemotherapy. The chemotherapy protocols employed included XELOX, consisting of capecitabine and oxaliplatin, and FOLFOX, comprising 5-fluorouracil, leucovorin or levoleucovorin, and oxaliplatin. The administration of PD-1/PD-L1 inhibitors was conducted as follows: Pembrolizumab (Merck & Co., USA) was administered at a dosage of 200 mg intravenously every three weeks; Sintilimab (Innovent Biologics, China) was also given at 200 mg intravenously every three weeks; Nivolumab (Bristol-Myers Squibb, USA) was administered at a dosage of 3 mg/kg intravenously every two weeks.

Text S3. CT image features

The following CT image features were evaluated:

(a) Tumor location: Categorized as cardia, body, antrum, or involving ≥2/3 of the stomach;

(b) Tumor size:

(i) Tumor length: Defined as the longest diameter of the tumor on its maximum cross-sectional plane in the portal venous phase, assessed using multi-planar reconstruction (MPR);

(ii) Tumor thickness: Defined as the maximum diameter perpendicular to the gastric wall on axial portal venous phase images;

(c) CT values, including plain, arterial, and portal venous phase CT values;

(d) Enhancement degree: Determined by the difference in HU between the portal venous phase and the plain scan. An enhancement value of ≤ 40 HU was classified as mild-to-moderate enhancement, while an enhancement value of > 40 HU was classified as marked enhancement;

(e) Enhancement pattern: Categorized as persistent, washout, or progressive;

(f) clinical staging: Assessed according to the 8th edition of the American Joint Committee on Cancer (AJCC) / Union for International Cancer Control (UICC) staging system.

Text S4. Image process and ROI segmentation

For the optimal visualization of gastric cancer lesions, portal venous phase CT images were utilized in this study for radiomics analysis. All patient images underwent processing using the 3D Slicer software (version 5.0.2, http://www.slicer.org). To enhance the robustness of radiomics features across varying scanning protocols, preprocessing was applied to all CT images. This included voxel resampling to dimensions of 1 mm × 1 mm × 1 mm, discretization of gray-scale values with a bin width of 25, and normalization of gray-scale values. Following preprocessing, the tumor region of interest (ROI) was manually delineated on the maximum transverse section of the tumor, following the lesion margin.

Text S5. Machine Learning Model Implementation and Hyperparameters

This document provides details on the R packages and hyperparameter settings used for each of the five machine learning models evaluated in this study. All models were built using the R software (Version 4.2.2). The predictor variables used for model construction were treatment cycle, CA72-4, and radscore. The binary outcome variable was “Subtype” (Response/Non-response).

formula：Subtype == "Response" ~ treatment cycle + CA72.4 + radscore.

The R packages used for constructing the machine learning models and the settings for hyperparameters are as follows:

Machine learning models	R Package	Hyperparameter setting
Logistic Regression	stats	glm(formula, data = train, family = "binomial")
eXtreme Gradient Boosting	xgboost	xgboost(data = train, label = train$Subtype, nrounds = 1000)
Support Vector Machine	e1071	svm(formula, data = train, kernel = 'radial', type = 'C-classification', probability = T)
Naive Bayes	e1071	naiveBayes(formula, data = train)
Random Forest	randomForest	randomForest(x = train[,features], y = train$Subtype, ntree = 100)

Supplementary Tables

Table S1 CT Imaging Protocol and Scan Parameters

Parameter	Center 1	Center 2
CT Scanner	Revolution CT, IQon Spectral CT	Revolution CT
Collimator Width	80 mm or 64 × 0.625 mm	80 mm
Tube Rotation Speed	0.5 s/rotation	0.5 s/rotation
Pitch	0.992:1 or 1.375:1	0.992:1
Field of View	500 × 500 mm	500 × 500 mm
Tube Voltage	120 kVp	120 kVp
Tube Current	Auto mA	Auto mA
Contrast Agent	Iohexol (350 mgI/ml), Ioversol (320 mgI/ml)	Ioversol (320 mgI/ml)
Total Contrast Dose	1.5 ml/kg	1.5 ml/kg
Contrast Injection Rate	3.0 ml/s	3.0 ml/s
Arterial Phase Acquisition Time	30 s	30 s
Portal venous phase Acquisition Time	70 s	60 s

Table S2 Comparison of AUC Values Among Different Machine Learning Models

Dataset	Model Comparison	*Z*	*P*
Training	Clinical vs. Radiomics	-7.431	＜0.001
	Clinical vs. Logistic	-7.998	＜0.001
	Logistic vs. XGB	-4.726	**＜0.001**
	Logistic vs. SVM	-0.073	0.941
	Logistic vs. RF	-4.626	**＜0.001**
	Logistic vs. NB	1.894	0.058
	XGB vs. SVM	4.641	**＜0.001**
	XGB vs.RF	3.360	**0.001**
	XGB vs.NB	4.997	**0.001**
	SVM vs. RF	-4.802	**＜0.001**
	SVM vs. NB	1.387	0.166
	RF vs. NB	4.453	**＜0.001**
Internal Validation	Clinical vs. Radiomics	-3.339	0.001
	Clinical vs. Logistic	-3.626	＜0.001
	Logistic vs. XGB	1.823	0.068
	Logistic vs. SVM	0.930	0.352
	Logistic vs. RF	0.221	0.825
	Logistic vs. NB	0.845	0.398
	XGB vs. SVM	-0.941	0.347
	XGB vs.RF	-1.508	0.132
	XGB vs.NB	-1.646	0.100
	SVM vs. RF	-0.581	0.561
	SVM vs. NB	-0.810	0.418
	RF vs. NB	0.044	0.965
External Validation	Clinical vs. Radiomics	-2.116	0.038
	Clinical vs. Logistic	-2.393	0.019
	Logistic vs. XGB	1.591	0.112
	Logistic vs. SVM	1.819	0.069
	Logistic vs. RF	0.885	0.376
	Logistic vs. NB	1.612	0.107
	XGB vs. SVM	0.078	0.938
	XGB vs.RF	-1.055	0.291
	XGB vs.NB	-0.712	0.477
	SVM vs. RF	-1.235	0.217
	SVM vs. NB	-0.934	0.350
	RF vs. NB	0.746	0.456

Supplementary Figures

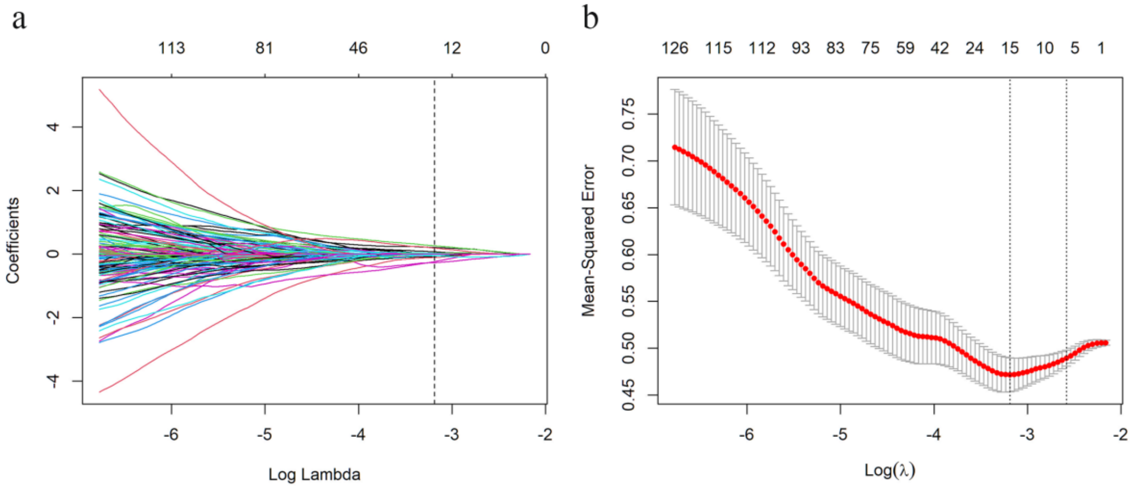

Figure S1 LASSO regression analysis. a: The LASSO coefficient profiles of the radiomics features. The top x-axis indicates the number of features, while the bottom x-axis represents the log-transformed lambda values. The y-axis shows the value of the regression coefficients; b: Tuning parameter (lambda) selection in the LASSO model using 10-fold cross-validation. The y-axis represents the Mean Squared Error (MSE), while the bottom and top x-axes show the log(lambda) values and the corresponding number of non-zero features, respectively. The two vertical dashed lines indicate the lambda.min (the lambda value that gives the minimum MSE) and the lambda.1se (the most regularized model such that the error is within one standard error of the minimum). Based on the optimal lambda.min value, a total of 15 radiomics features with non-zero coefficients were selected.
